# Formation of Nanocomplexes between Carboxymethyl Inulin and Bovine Serum Albumin via pH-Induced Electrostatic Interaction

**DOI:** 10.3390/molecules24173056

**Published:** 2019-08-22

**Authors:** Guiying Huang, Jun Liu, Weiping Jin, Zihao Wei, Chi-Tang Ho, Suqing Zhao, Kun Zhang, Qingrong Huang

**Affiliations:** 1 Faculty of Chemical Engineering and Light Industry, Guangdong University of Technology, Guangzhou 510006, Guangdong, China; 2 Department of Food Science, Rutgers University, 65 Dudley Road, New Brunswick, NJ 08901, USA; 3 College of Light Industry and Food Science, Zhongkai University of Agriculture and Engineering, Guangzhou 510225, Guangdong, China; 4 College of Food Science and Engineering, Yangzhou University, Yangzhou 225127, Jiangsu, China; 5 College of Food Science and Engineering, Wuhan Polytechnic University, 68 Xuefu South Road, Wuhan 430023, Hubei, China; 6 College of Chemistry and Environmental Engineering, Wuyi University, Jiangmen 529020, Guangdong, China

**Keywords:** carboxymethylated inulin, nanocomplexes, turbidimetric titrations, isothermal titration calorimetry, ζ-potential measurements, atomic force microscopy

## Abstract

As a functional polysaccharide, inulin was carboxymethylated and it formed nanocomplexes with bovine serum albumin (BSA). The success of obtaining carboxymethyl inulin (CMI) was confirmed by a combination of Fourier transform Infrared (FT-IR), Raman spectroscopy, gel permeation chromatography (GPC), and titration. The effects of pH and ionic strength on the formation of CMI/BSA nanocomplexes were investigated. Our results showed that the formation of complex coacervate (pH_φ1_) and dissolution of CMI/BSA insoluble complexes (pH_φ2_) appeared in pH near 4.85 and 2.00 respectively. FT-IR and Raman data confirmed the existence of electrostatic interaction and hydrogen bonding between CMI and BSA. The isothermal titration calorimetry (ITC) results suggested that the process of complex formation was spontaneous and exothermic. The complexation was dominated by enthalpy changes (∆Η < 0, ∆S < 0) at pH 4.00, while it was contributed by enthalpic and entropic changes (∆Η < 0, ∆S > 0) at pH 2.60. Irregularly shaped insoluble complexes and globular soluble nanocomplexes (about 150 nm) were observed in CMI/BSA complexes at pH 4.00 and 2.60 while using optical microscopy and atomic force microscopy, respectively. The sodium chloride suppression effect on CMI/BSA complexes was confirmed by the decrease of incipient pH for soluble complex formation (or pH_c_) and pH_φ1_ under different sodium chloride concentrations. This research presents a new functional system with the potential for delivering bioactive food ingredients.

## 1. Introduction

Polysaccharides and proteins are biopolymers that are commonly used in food processing. Generally speaking, polysaccharides can be used to stabilize emulsions and suspensions, as well as to improve the thickening and gelling properties of food products. Proteins play a significant role in the sensory attributes of food, such as texture, flavor, color, and appearance [1]. Polysaccharides and proteins are often applied together to modify the structure and stability of food [2,3]. As the simultaneous addition of polysaccharides and oppositely charged proteins in certain specific pH ranges can induce electrostatic interaction and cause either soluble or insoluble complex formation, which can not only be used to enhance the quality of food, but also participate in nutraceuticals and flavors delivery, increasing interest arises for the understanding of phenomena that are associated with these charge-charge interactions [4,5,6,7,8,9,10,11,12,13].

Either soluble or insoluble complexes that are formed by interactions between polysaccharides and proteins result from the competition between electrostatic attraction, which promotes association and entropy effects that assist dispersion [14,15]. Therefore, many physicochemical parameters, including the type and charge density of polysaccharides and proteins, solution pH value, ionic strength, and polysaccharide/protein ratio can affect the formation of protein/polysaccharide complexes. As basic ingredients in the systems, proteins can be self-assembled and further form complexes with anionic or cationic polysaccharides that are induced by pH [10,16]. In contrast, polysaccharides show more flexible functions in the system. On one hand, polysaccharides can be added to enhance the self-association of protein [17]. On the other hand, ion modified polysaccharides diversify protein/polysaccharide complexes, such as carboxyl methyl cellulose/whey protein complexes and carboxyl methyl dextran/whey protein complexes [18,19,20,21,22,23,24]. 

At the isoelectric point of a protein, the interaction between protein “charged patches” (charge distribution) and oppositely charged group of a polysaccharide is mainly determined by electrostatic interaction [25,26]. Phase diagrams need to be studied to better understand the interactions between polysaccharides and oppositely charged proteins since phase change occurs under different pH values. For the mixture of a weak negatively charged polysaccharide and a protein, the typical phase diagram is separated by three phase boundaries (pH_c_, pH_φ1_, and pH_φ2_) into four regions [12]: (1) two soluble polymers coexist with no complex formation at pH above pH_c_; (2) when pH is below pH_c_, positive charged patches of the protein interact with negatively charged polysaccharides to form soluble nanocomplexes; (3) when the pH is further reduced to pH_φ1_, insoluble complexes are generated; and, (4) when the pH continues to decrease to pH_φ2_, the interaction between weakly charged polysaccharide and protein decreases, and the insoluble complexes are suppressed into disconnected polysaccharide and protein molecules [8,27]. During this period, soluble complexes can form at pH_c_ and pH_φ2_ [28].

**Figure 1:**
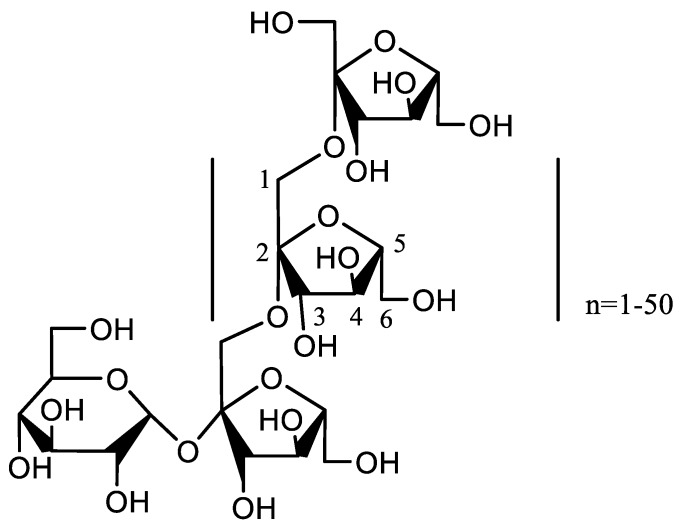
Chemical structure of inulin.

Inulin is mainly composed of 2→1 linked β-D-fructofuranosyl units, which is shown in Figure 1 [29]. Inulin exists in various plants, especially rich in the tubers of Jerusalem artichoke and Chicory [30]. As a prebiotic, inulin will not raise the glucose and insulin levels in the blood of human body, and it can only be decomposed by bacteria into carbon dioxide and short chain fatty acids in the large intestine [31]. In food industry, inulin is applied to protect protein and some liposomes from damage during food processing, or as a fat replacer being used in healthy foods [32,33,34]. In the pharmaceutical industry, inulin is used as a well-known adjuvant to enhance vaccine efficacy against influenza [35,36]. To expand the application of inulin, carboxyl methylation is an effective modification method with multiple advantages [37]. Carboxyl methyl inulin (CMI) is non-toxic, and it has high solubility, biocompatibility, and biodegradability [38,39,40]. CMI can not only improve the absorption of calcium in organism, but it can also reduce the cytotoxicity of functional substances [41]. Theoretically, CMI is an anionic polysaccharide that is suitable for the complex formation with cationic protein as a type of functional biopolymer complexes for food ingredients, fat replacers, package, and drug delivery [42,43]. CMI binding with protein can enlarge the types of functional compounds multifunctional applications in human nutrition. However, until now, no report is available regarding the assembly of nanocomplexes from CMI and proteins.

In this study, the complexes formation of CMI and bovine serum albumin (BSA, functional globular protein for human body) induced by electrostatic interaction were investigated. The effects of CMI/BSA ratios and ionic strengths on complexes structure were evaluated by an array of characterization tools. Turbidimetric titration method was applied to confirm the attractive interaction and determine the phase diagrams between CMI and BSA. Dynamic light scattering was used to measure the sizes of soluble complexes in the onset and suppression period of insoluble complexes. Atomic force microscopy was further used to observe the structure and morphology of the complexes. Our results presented a clear pattern of the formation of complexes between CMI and BSA via pH-induced electrostatic interaction.

## 2. Results

### 2.1. Characteristics of CMI

#### 2.1.1. FT-IR Spectra of CMI

**Figure 2:**
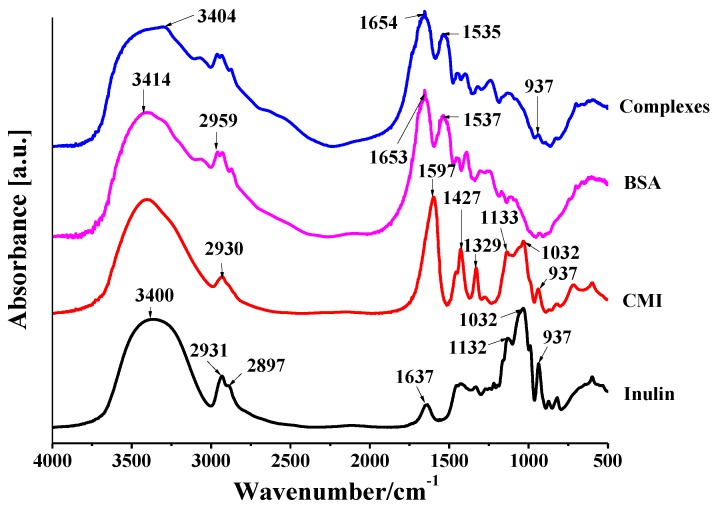
Infrared spectra of inulin, carboxyl methyl inulin (CMI), bovine serum albumin (BSA), and CMI/BSA complexes.

In the IR spectra of inulin (Figure 2), the characteristic absorption band of inulin at 3400 cm^−1^ (O-H stretching of glucose and fructose units), 2931 cm^−1^ and 2897 cm^−1^ (C-H stretching), 1637 cm^−1^ (hydroxyl bending), and 1032 cm^−1^ and 937 cm^−1^ (C-O-O stretching) were observed. In the spectra of CMI (Figure 2), the intensity of hydroxyl stretching in CMI was lower than that in inulin, which indicated that the amount of O-H in inulin backbone decreased after carboxymethylation. Two significant characteristic peaks appeared at 1597 cm^−1^ and 1427 cm^−1^, which were attributed to asymmetric and symmetric stretching of the COO^−^, respectively. Hence, hydroxyl groups of inulin were substituted by carboxylate groups, and the successful synthesis of CMI from inulin was confirmed. Additionally, similar shapes of peaks between 1200–900 cm^−1^ in the IR spectra of inulin and CMI showed that the synthesized product still maintained the backbone integrity of inulin [39].

#### 2.1.2. Raman Spectra of CMI

**Figure 3:**
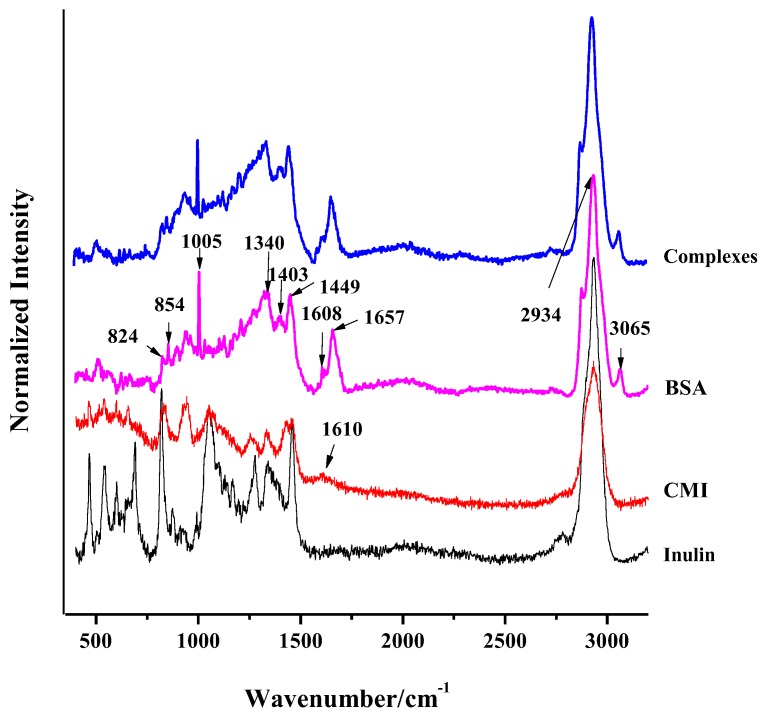
Raman spectra of inulin, carboxyl methyl inulin (CMI), BSA, and CMI/BSA complexes.

Raman spectroscopy is non-destructive to the samples and it can collect more information of surface structure and backbone of the samples. The characteristic absorption bands of inulin at 2934 cm^−1^ (C-H stretching), 1260 cm^−1^ (C-O-H stretching and bending vibrations), 870–890 cm^−1^ (C-C or C-O vibrations coupled with the C-H mode of the anomeric carbon of β-conformers), and below 700 cm^−1^ (skeleton and complex ring vibrations) are shown in Figure 3. For CMI, a new broad band at 1610 cm^−1^ was observed. This band is mainly ascribed to substituted carboxymethylate groups, rather than native carboxyl groups from polysaccharides themselves. The COO^−^ stretching vibration at peak 1425 cm^−1^ was also observed [44]. Hence, the successfully carboxymethylation of inulin was confirmed from another perspective. The bands at 700–500 cm^−1^ were assigned to be the crystalline region. The crystalline region of CMI was found to be lower than that of inulin, suggesting that inulin’s crystalline structure was altered after carboxymethylation. This can partially explain why CMI has a higher solubility than inulin. Although the intensity of peaks at 1500–800 cm^−1^ changed, their shapes still maintained.

#### 2.1.3. Degree of Substitution (DS) and Molecular Weight (Mw) of CMI

**Figure 4:**
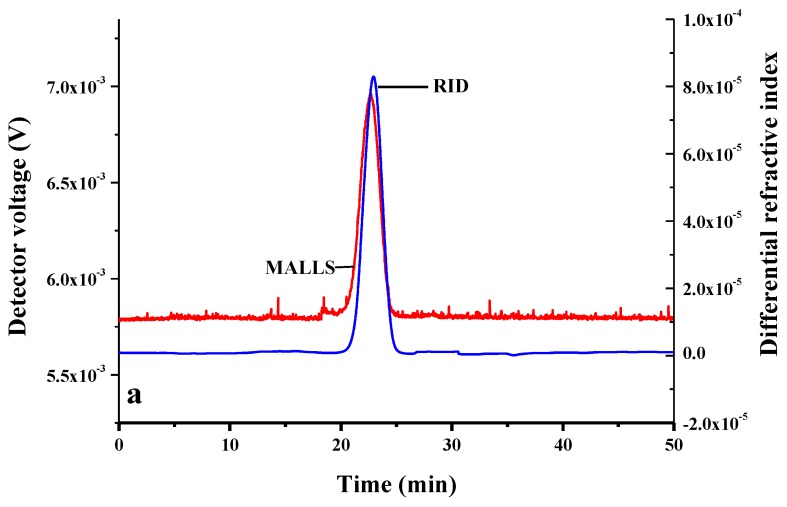

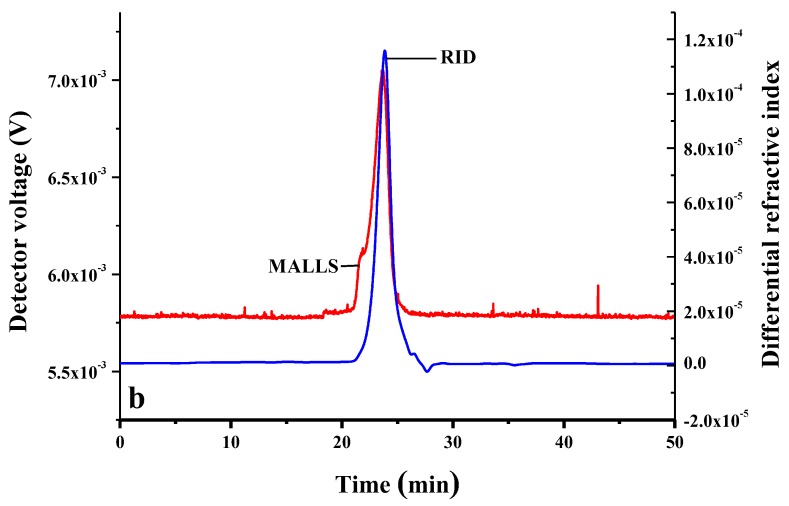
Gel permeation chromatography (GPC) chromatograms of inulin (**a**) and CMI (**b**).

The molecular weight of CMI was determined while using gel permeation chromatography multi-angle laser light scattering (GPC-MALLS-RID). GPC chromatograms of CMI with a sharp peak are shown in Figure 4a. The value of dn/dc (mL/g) and polydispersity (Mw/Mn) of CMI were 0.13 and 1.11, respectively, which indicated that CMI was monodisperse [45,46]. When compared with GPC chromatograms of inulin in Figure 4b, the peak of CMI had shorter retention time, which suggested that the molecular of CMI was higher than inulin (2.70 × 10^3^ ± 96 Da). This increment was attributed to the success of grafting of carboxy methyl groups in the inulin backbones [47,48]. The molecular weight of CMI was calculated as 3.93 × 10^3^ ± 31 Da. Additionally, DS of CMI was determined to be 0.49 ± 0.0022 while using titration methods. 

### 2.2. Effect of pH on the Formation of CMI/BSA Complexes

**Figure 5:**
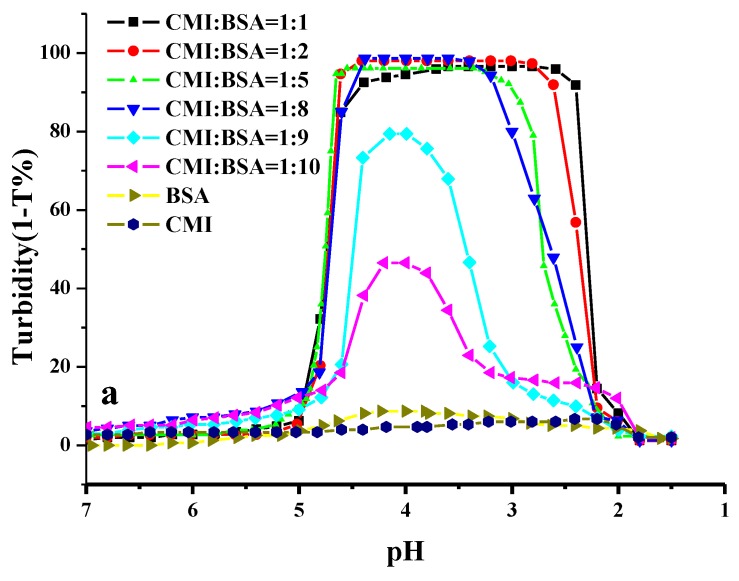

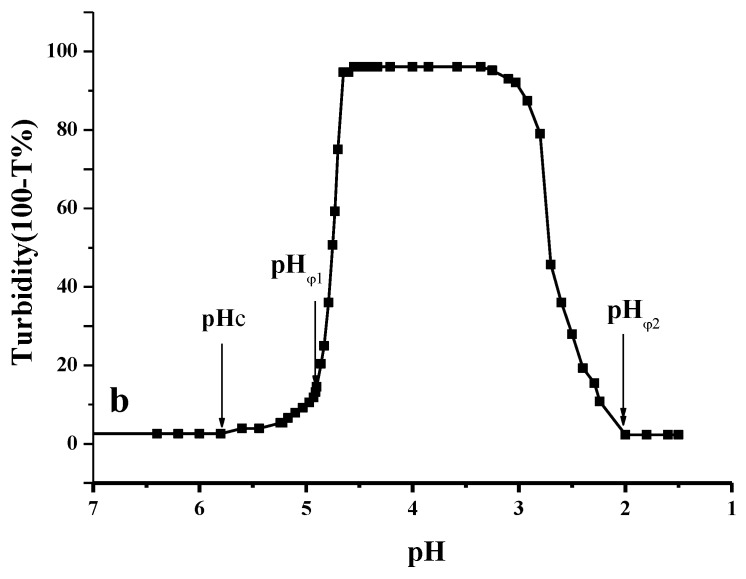
The turbidimetric titration curves of CMI/BSAmixtures as a function of pH at different ratios (**a**) and at CMI/BSA ratio (r) = 1:5 (**b**).

Turbidimetric titration method was used to confirm complexation between CMI and BSA since the change in turbidity is mainly caused by the formation and dissociation of complexes [49]. Turbidity of CMI/BSA complexes was investigated in the pH range of 7.0 to 1.5 by adjusting pH with HCl. Figure 5a showed the turbidimetric titration curves of the CMI, BSA, and CMI/BSA at different ratios. A similar shape of curves was found in preliminary work of our lab in pectin/BSA complexes [17]. When the concentration of BSA increased, the highest turbidities were observed at CMI/BSA ratio of 1:1 to 1:5, exhibiting that a strong reaction existed between negative carboxyl groups of CMI and positive amino groups of BSA [13,50]. The content of BSA increase barely shifted the pH_c_, pH_φ1_, and pH_φ2._ During the titration period, most stability of turbidities near pH_c_ was observed at 1:5 ratio. The turbidities decreased when the ratio increased from 1:8 to 1:10. This finding indicated that repulsive force between BSA molecules took effect. The effect in complexes of pH on CMI/BSA mixture (*w*/*w* = 1:5) in 10 mM NaCl solution was selected for further study in Figure 5b. During acidification, the curve of CMI solution was almost flat within the investigated pH range, which indicated that the turbidity of CMI solution was barely affected by pH. Similar phenomenon was also observed in the curve of BSA solution, except for the minor increase in turbidity within the pH range of 4.9 to 3.5, which is close to the isoelectric point (pI = 4.7) of BSA. The slight increase in turbidity suggested that the small degree of aggregation of BSA occurred in pH value around pI.

In contrast to CMI or BSA solutions alone, the turbidity of CMI/BSA mixture started to slightly increase at pH 5.75. When the pH value reached to 4.85, the turbidity abruptly rose to a high value around 97%, this value was retained until pH decreased to ~3.1, and then slowly declined from pH 3.30 to 2.00. Therefore, pH values of 5.75, 4.85, and 2.00 could be identified as pH_c_ (incipient of soluble complex formation), pH_φ1_ (global phase separation), and pH_φ2_ (dissolution of complex), respectively. Accordingly, the turbidity curve was divided into four regions by the three critical pH values. At pH > pH_c_ (the first region), there was no change in the masses or sizes in CMI and BSA, and CMI and BSA could coexist in the solution as monomers. When pH approached pH_c_, the turbidity of the mixture solution began to increase slowly, which suggested that the formation of soluble CMI/BSA complexes was initiated at pH_c_. At pH values between pH_c_ and pH_φ1_ (the second region), soluble complexes formed at pH values above pI as precursors of insoluble complexes. A similar phenomenon was also observed in other studies [27,51,52,53]. When the pH value was nearby the pI of BSA, carboxylate groups in CMI could bond to the positive domain of BSA due to charge anisotropy [25]. Additionally, the BSA model at pH 5.0 (higher than pI) in the scheme came from the Monte Carlo research in our lab [54].

When pH value declined from pH_φ1_ to pH_φ2_ (the third region), drastically decreased turbidity was observed, along with the occurrence and termination of phase separation. At least two kinds of morphologies of complexes appeared, due to obvious change in mass and size at this specified region, which will be discussed in Section 3.5. Since the turbidity curve showed the existence of the soluble complexes in the first region, “Veis” aggregate model was applied to describe the phase separation in CMI/BSA mixture solution [25]. There were two steps leading to the phase separation in the CMI/BSA system. First, complexes of low configurational entropy formed due to the strong interaction between oppositely charged CMI and BSA via electrostatic force until charge neutrality. Subsequently, the rearrangement of these complexes to form larger insoluble complexes and phase separation occurred. Without intercomplexes repulsion of CMI/BSA, the second step was usually in progress, along with non-electrostatic interactions, such as hydrogen bonding or hydrophobic force. At last, the CMI/BSA complexes were completely suppressed when the pH was lower than pH_φ2_, when the amount of charges of CMI approached zero. In this case, the electrostatic interaction between CMI and BSA became minimal.

### 2.3. Effect of Ionic Strength on the Formation of CMI/BSA Complexes

Ionic strength, which can be described in the form of salt concentration of sodium chloride, is a critical parameter that influences the formation of complexes. By adding salt into the oppositely charged CMI and BSA mixed solutions, monovalent ions can screen either electrostatic attraction or repulsion, leading to the shifts of pH_c_, pH_φ1_, and pH_φ2_, as well as the enhancement and suppression of the formation of insoluble complexes [27]. In this study, the turbidity change of CMI/BSA mixture under a range of pH value was observed in the absence and presence of salt. Different salt concentrations (C_NaCl_), including 0, 10, 50, 100, and 200 mM, respectively, were used in the CMI/BSA (CMI: BSA = 1:5) mixed systems, and the pH value was adjusted from 7.0 to 1.5 by using 0.1 M HCl. 

**Figure 6:**
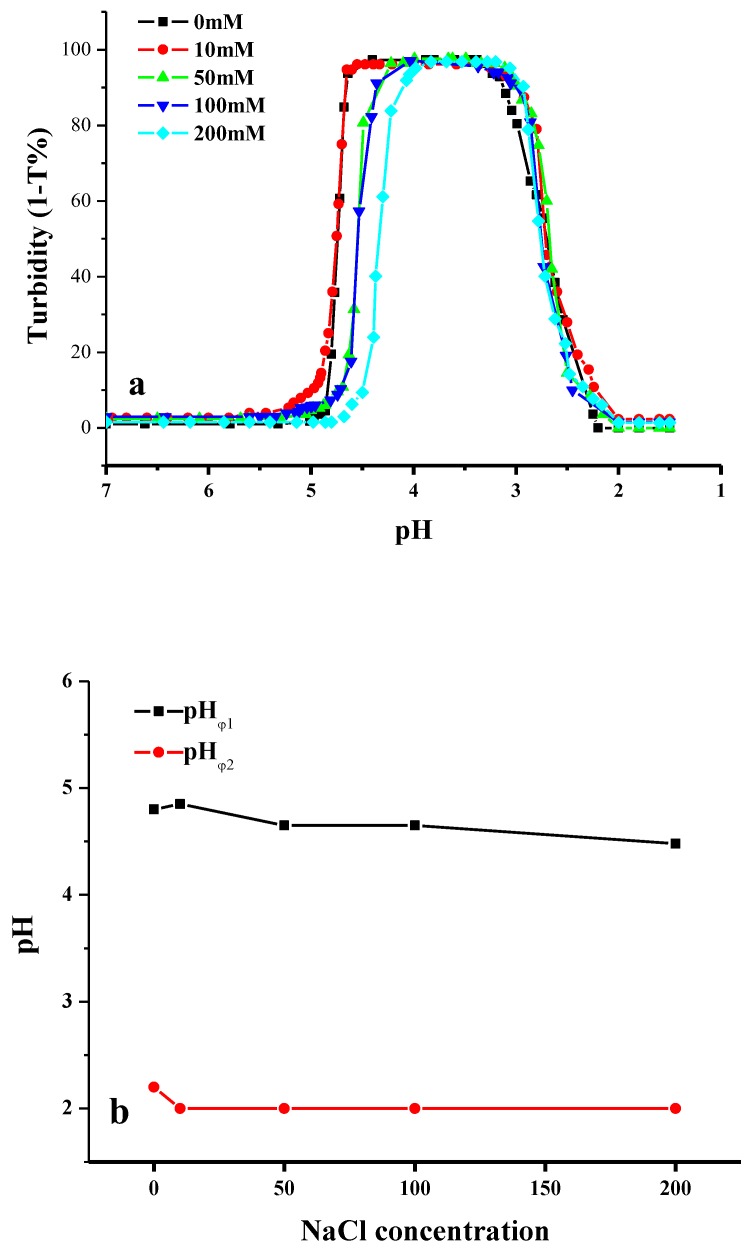
The turbidimetric titration curves of CMI/BSA mixtures as a function of pH at different C_NaCl_ at CMI/BSA ratio (r) = 1:5 (**a**) and the variation of pH_φ1_ and pH_φ2_ as a function of C_NaCl_ (**b**).

As shown in Figure 6a,b, there were two phenomena presented in the turbidity curves under different salt concentrations. When comparing the salt free system (C_NaCl_ = 0 mM) with the low salt system (C_NaCl_ = 10 mM), pH_φ1_ rose from 4.80 to 4.85, and pH_φ2_ decreased from 2.20 to 2.00. The insoluble complexes area distinctly increased. However, the width of the insoluble complexes area decreased with the increase of ionic strength. The further addition of salt caused a pH_φ1_ value change from 4.85 to 4.40. pH_φ2_ values of all the CMI/BSA mixtures were less dependent upon salt concentration. Salt enhancement and suppression effects were observed at the critical NaCl concentration of 10 mM for CMI/BSA mixtures. This result is similar to those that were reported by Dubin et al. and Huang et al. [27,51]. When C_NaCl_ < 10 mM, the addition of a small amount of salt was mainly to screen electrostatic repulsion between the negative charged patches that were caused by anisotropy of BSA and same charge of CMI under the pH value higher than pI of BSA in the second region (from pH_c_ and pH_φ1_). When C_NaCl_ > 10mM, the decreased tendency of pH_c_ and pH_φ1_ with the increase of ionic strength illustrated that the screening of the attraction between CMI and BSA took place due to the presence of ions [51]. Against the screening effect, the higher ionic strength required more positive charges of BSA for the electrostatic interaction with CMI, thus the onset of soluble complexes might occur at a pH value much lower than the pI of BSA under a relatively high salt concentration. Furthermore, screening of the charges also reduced the range of complex formation, which was also revealed by Weinbreck’s paper [12]. Moreover, this was the salt suppression effects. However, the similar shape of titration curves from pH 3.30 to 2.00 under different salt concentrations (0, 10, 50, 100, and 200 mM) indicated that the dissociation of insoluble complexes was mainly caused by the charge reduction of CMI induced by pH, rather than ionic strength. 

### 2.4. Characteristics of CMI/BSA Complexes

#### 2.4.1. FT-IR Spectra of CMI/BSA Complexes

The IR spectra of BSA and CMI/BSA complexes are shown in Figure 2. The characteristic peaks of BSA at 1500–1660 cm^−1^ attributed to amide I and amide II vibrations. A broad band around 3414 cm^−1^ assigned to O-H stretching vibrations. A peak at 2959 cm^−1^ was ascribed to C-H vibration. In spectra of complexes, the characteristic peaks of BSA maintained, suggesting that CMI and BSA were non-covalent combination. A shift of O-H stretching from 3414 to 3404 cm^−1^ suggested the existence of hydrogen bonding between CMI and BSA. Moreover, the change of shapes of peaks at 1500–1660 cm^−1^ of amide I and amide II vibrations assigned to physical interaction between CMI and BSA [26,55]. When compared with the spectra of CMI, a clear decrease of peaks at 1597 and 1427 cm^−1^ (COO^−^ stretching) and 1032 and 937 cm^−1^ (C-O-C stretching) occurred in the spectra of the complexes. This finding suggested that the CMI/BSA complexes were formed via electrostatic interaction. 

#### 2.4.2. Raman Spectra of CMI

Raman spectra of BSA and CMI/BSA complexes are exhibited in Figure 3. The characteristic bands of BSA at 508 cm^−1^ (S-S stretching vibrations), 824 and 854 cm^−1^ (tyrosine doublet), 936–990 cm^−1^ (amide II band), 1005 cm^−1^ (phenylalanine), 1340 cm^−1^ (C-H bending or tryptophan), 1452 cm^−1^ (C-H bending), 1657 cm^−1^ (amide I band), 2934 cm^−1^ (C-H stretching of various amino acids), and 3063 cm^−1^ (=C-H stretching of aromatic amino acids) could be observed in the curve, while insignificant peak at 750 cm^−1^ (tryptophan) suggested that trp might be buried in the protein interior [56]. The curve of complexes was similar to that of BSA, which indicated that BSA molecules were on the surface of complexes, while CMI was inside. Minor change of secondary structure as compared to BSA suggested that no covalent bonding existed between BSA and CMI [57]. The intensity ratio of 824 and 854 cm^−1^ (tyrosine doublet) reflected tyrosine side chain of BSA in the complexes. *I*_854_/*I*_824_ was reduced from 1.31 in the BSA curve to 1.14 in complexes [58]. The decrease exhibited that the Tyr residues as a strong hydrogen bond were inside the complexes, rather than on the surface. This suggested the existence of hydrogen bonding between BSA and CMI, which agreed with the observation of IR spectra. The peak at 750 cm^−1^ remaining almost unchanged in complexes was probably attributed to the hydrophobic interaction between BSA molecules, which might arise from their aggregation. 

### 2.5. Isothermal Titration Calorimetry (ITC) of CMI/BSA Interaction

**Figure 7:**
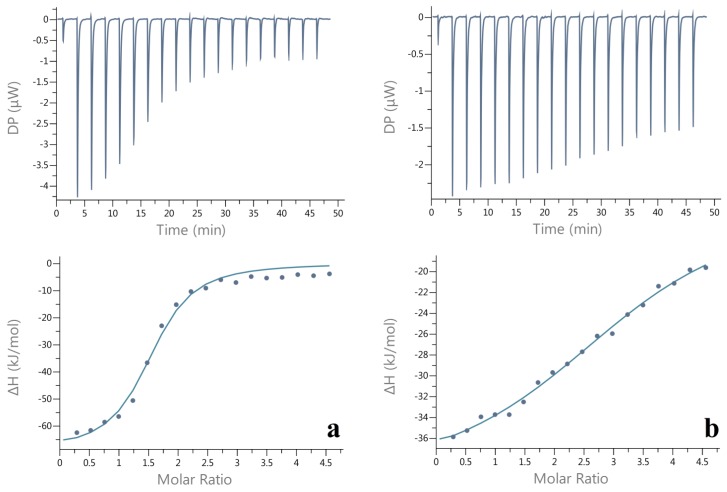
Thermogram (upper panel) and binding isotherms with theoretical fits (lower panel) of the titration of CMI into BSA at pH 4.00 (**a**) and pH 2.60 (**b**), respectively (C_NaCl_ = 10mM).

Isothermal titration calorimetry (ITC) is a direct and effective technique to quantitatively collect thermodynamic parameters in polysaccharide/protein interaction. The critical advantage of ITC is that the measurement will not be affected by different phase transitions that couple with the aggregation of molecules [24]. Hence, ITC measurement was employed for the condition of energy changes in the formation of insoluble and soluble complexes between CMI and BSA. The experiments were performed at pH 4.00 and pH 2.60, respectively. The heat flow versus time profiles that result from titration of BSA with CMI in a reaction cell and detecting the heat absorbed or released. The mass concentrations of CMI and BSA were set as 0.12% and 0.1% (*w*/*v*), respectively. Additionally, the molar concentration ratio of CMI (average molecular weight detected by GPC) and BSA were calculated. Owing to the structure of CMI and BSA and only one inflection points in the binding isotherm (bottom panel in Figure 7), thermodynamic parameters, including binding stoichiometry (N), entropy (ΔH), enthalpy changes (ΔS), and free energy (ΔG) were calculated by iterative curve fitting of the binding isotherms while using a “one set of sites” model [59].

The thermodynamic injections of CMI solution into BSA solution were set at 2 μL every 150 s under pH 4.0 and pH 2.60, respectively, with C_NaCl_ of 10 mM. As the thermodynamic graph shown in Figure 7, a sequence of strong successive exothermic peaks of decreasing intensity was initially observed. With more injections, the released binding energy continuously declined and then reached to a state of thermodynamic stability after the 12nd and 14th injection, respectively. Similar results were observed in lactalbumin/chitosan complexes, gelatin/sodium carboxymethyl cellulose complexex, and ovalbumin/carboxymethylcellulose complexes [28,60,61].

Binding stoichiometry (N), affinity constant (Ka), enthalpy (ΔH), entropy (TΔS), and Gibbs free energy change (ΔG) of the titration of CMI into BSA at pH 4.00 were 1.50 ± 0.09, (7.19 ± 1.25) × 10^5^ M^−1^, −69.50 ± 5.63 kJ/moL, −36.00 kJ/moL, −33.50 ± 5.63 kJ/moL. These thermodynamic parameters of the titration of CMI into BSA at pH 2.60 were 3.28 ± 0.33, (1.10 ± 0.34) × 10^5^ M^−1^, −26.9 ± 3.81 kJ/moL, 1.96 kJ/moL, −28.86 ± 3.81 kJ/moL. Negative values of ΔG suggest that spontaneous interactions between CMI and BSA occurred at pH 4.00 and pH 2.60. On one hand, the interaction at pH4.00 was dominated by enthalpy changes (∆Η < 0, ∆S < 0), owing to the electrostatic interaction and hydrogen bonding (Figure 2 and Figure 3). On the other hand, the interaction at pH 2.60 was driven by both enthalpy and entropy, showing that the interaction was a combination of changes in the attractive interactions between the charged groups (COO^−^ and NH_2_^+^) and entropy of electrostatic repulsion interactions between positive BSA molecules (∆Η < 0, ∆S > 0). The binding stoichiometry (N) of CMI/BSA complexes at pH 2.60 were greater than those at pH 2.60 exhibited that BSA molecules combined with more CMI chains at lower pH [13]. 

### 2.6. Morphology of CMI/BSA Complexes

**Figure 8:**
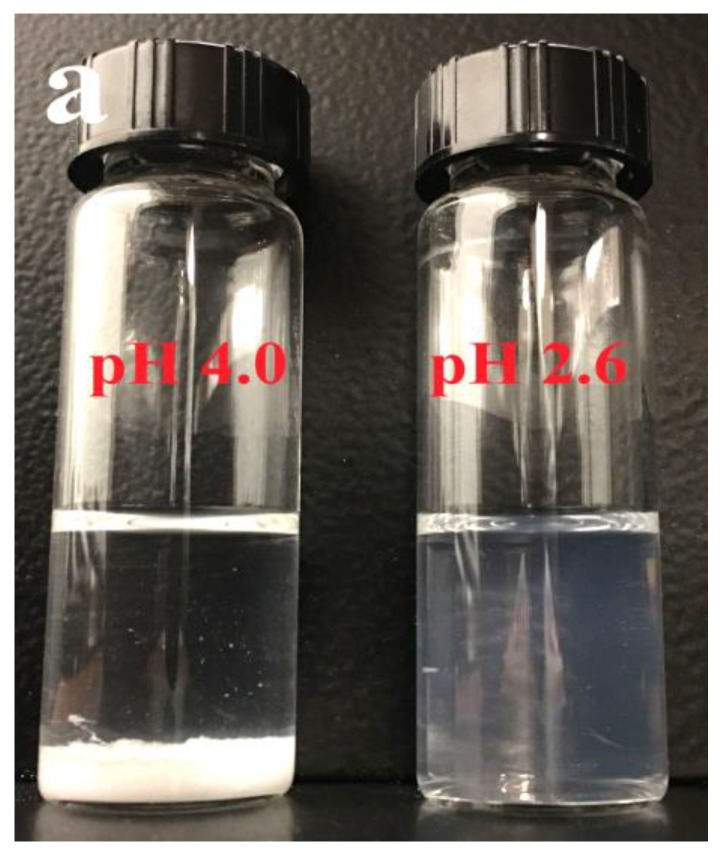

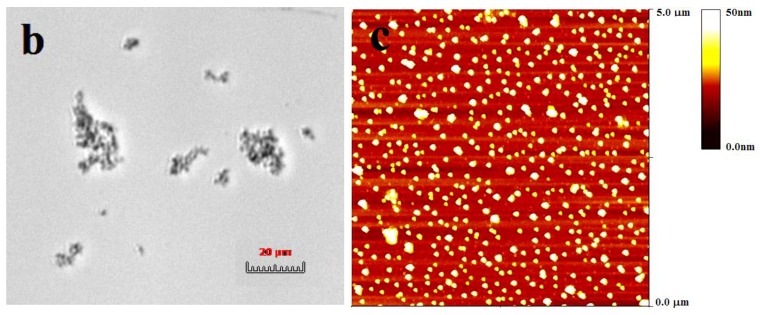
Optical picture of vials containing CMI/BSA complexes prepared at pH = 4.00 (left) and 2.60 (right) after storage at room temperature for one week (**a**). Optical microscopic images of insoluble complexes of CMI/BSA at C_NaCl_ = 10mM at pH = 4.0 and ratio (r) = 1:5 (**b**). Atomic force microscopic height (AFM) image of CMI/BSA complexes with CMI/BSA ratio (r) = 1:5, C_NaCl_ = 10 mM and pH = 2.6 (**c**).

Dynamic light scattering (DLS), atom force microscopy (AFM), optical microscopy, and ζ-potential experiments were used to characterize the morphology of CMI/BSA complexes that formed in third region, since two kinds of specific CMI/BSA complexes existed with CMI/BSA ratio as 1:5, under pH values from pH_φ1_ to pH_φ2_. Two key pH values of 4.00 and 2.60 were chosen to compare the morphologies of the complexes. The main difference between pH-induced insoluble complexes (i.e., at pH 4.00) and soluble complexes (i.e., at pH 2.60) can be better illustrated in Figure 8a. At pH = 4.00, a clear global phase separation was observed (left image). However, at pH = 2.60, the complex solution was homogeneous and translucent (right image).

As presented in Figure 8b, the shapes of the insoluble CMI/BSA complexes were irregular schistose. The sizes of insoluble complexes ranged from 1 to 50 μm. Rounded edges, tight and compact network-like structures of the complexes formed under 10 mM salt concentration, and pH 4.00 could be observed.

At pH 2.60, nanocomplexes were obtained under different salt concentrations with sizes between 140 nm and 350 nm (Table 1). The polydispersity index (PDI) was defined as a way to assess polydispersity from the DLS results. Higher PDI values were found to correlate with higher ionic strength. This indicated that low salt concentration was favorable for the formation of uniform nanocomplexes. 

ζ-potential was measured to reveal the surface charge of the complexes. The result of ζ-potential accompanied with DLS and AFM could reveal the structure and surface charge of nanoparticles under a specific pH value [62]. The formation of the nanocomplexes was CMI coated by BSA since the positively charged nanocomplexes had been detected under 0, 10, and 50 mM salt concentrations. Tainaka’s model was used to explain the phenomenon [63]. As discussed in Section 3.2, insoluble complexes were mainly induced by attractive forces among aggregates of electrostatic neutrality. When pH continued to decrease to 1.5, the charge density of weak-polyelectrolyte CMI also decreased to approach zero. The weakened attraction between CMI and BSA led to the dissolution of insoluble complexes. At the same time, electrostatic repulsion force by positively charged BSA played a dominant role in the solution. The positive surface charges stabilized the nanocomplexes with BSA surrounding the surface at around pH 2.50. It was believed that positively charged nanocomplexes were the intermediates that formed between the electroneutral insoluble complexes. They would separate into CMI and BSA monomers after the complete dissociation of complexes. AFM image showed the shape of CMI/BSA nanocomplexes formed at pH 2.60 and 10 mM salt concentration (Figure 8c). Section analysis was used to analyze the sizes of air-dried nanoparticles [64,65]. The surface distance, horizontal distance, and vertical distance were 104.42, 97.656, and 24.661 nm, respectively. Therefore, the nanocomplexes were prolate sphere like nanoparticles, and were homogeneously dispersed. After one-week storage at room temperature, the sizes of the nanoparticles that were detected by DLS remained the same as before. 

## 3. Experimental Section

### 3.1. Materials

Inulin was extracted from Jerusalem artichoke and then purified in the Institute of Natural Medicine and Green Chemistry of Guangdong University of Technology (Guangzhou, China). Bovine serum albumin (BSA, >98%) was purchased from Sigma Chemical Co. (St. Louis, MO, USA). Isopropyl alcohol, analytical grade sodium chloride (NaCl), sodium hydroxide (NaOH), and hydrochloric acid (HCl) were purchased from Fisher Scientific (Waltham, MA, USA). Monochloroacetic acid (99%) was purchased from Sigma Chemical Co. (St. Louis, MO, USA). Milli-Q distilled water (18.3 Ω) was used in all of the experiments. 

### 3.2. Synthesis of CMI

CMI was synthesized according to the previously published method for carboxymethylated chitosan, with some modifications [66]. Inulin powder (10 g) was added into 100 mL of isopropyl alcohol and the mixture was stirred under magnetic stirring at room temperature for 3 h. Subsequently, 10 g NaOH was slowly added into the stirred mixture over a period of 30 min. The alkaline mixture was stirred for additional 30 min. Subsequently, monochloroacetic acid (40 g) was intermittently added in five equal portions within 5 min. The final mixture was heated at 60 °C for 12 h and then dialyzed in distilled water using a 500 Da molecular weight cut-off membrane for 72 h. The resulting solution was freeze-dried and reserved in a drying tower.

### 3.3. Characterization of CMI

Attenuated total reflectance Fourier transform Infrared (FT-IR) spectra were recorded on a Nicolet iS50 FT-IR spectrometer (Thermo Fisher Scientific Inc., Waltham, MA, USA) in the wave number range of 4000–400 cm^−1^. Raman spectra were obtained from LabRam HR800 with a helium-neon laser source providing radiation at 632.8 nm, as previously described [67]. The molecular weight distribution of CMI was analyzed by using gel permeation chromatography multi-angle laser light scattering (GPC-MALLS-RID, Wyatt Technology Corporation, CA, USA), with a Superose 6 10/300GL column (GE Healthcare, USA), according to Jin’s methods [68]. GPC was equipped with the column of OHpak SB-806 HQ, the DAWN HELEOS multi-angle light scattering detector (658 nm) and an Optilab rEX refractometer. Inulin powder was dissolved in the eluent (100mM NaCl aqueous solution) and then filtered through 0.22 mm Millipore membrane.

Degree of substitution (DS) of CMI was determined while using titration. CMI of 0.2 g was suspended in 15 mL of 2 M HCl solution containing 70% (*v*/*v*) of methanol. The mixture was stirred at room temperature for 1.5 h. Afterwards, the dispersion was washed several times with 95% (*v*/*v*) ethanol, filtered, and freeze-dried. Subsequently, the dried sample was redissolved in 10 mL of 0.5 M NaOH solution with stirring for 1.5 h. The resultant reaction mixture was finally titrated with 0.5 M HCl solution using phenolphthalein as the indicator. A blank test was conducted by using native inulin. The percentage of carboxymethyl group (*C*_a_) and the degree of substitution of CMI was calculated by the following equations:

(1) $$C_{a}\left(\%\right)=\frac{\left(V_{c}-V_{0}\right)\times 10^{-3}\times M_{c}\times 59\times 100}{W_{c}}$$

(2) $$\mathrm{Degree}\;\mathrm{of}\;\mathrm{substitution}=\frac{162\times C_{a}}{59\times 100-\left(59-1\right)\times C_{a}}$$

where *V*_c_ is the volume of 0.5 M HCl used for titration of native starch, *V*_0_ is the volume of 0.5 M HCl used for titration of CMI, *M*_c_ is the molarity of HCl solution, *W*_c_ is the weight of CMI, 59 is the molecular weight of carboxy methyl group, and 162 is the molecular weight of the fructose anhydride unit.

### 3.4. Preparation of CMI and BSA Mixed Solutions

To detect the effect of pH and ratio value on complexes, the concentration of CMI was fixed at 0.1% *w*/*v*, and the stock solutions were used to prepare different ratios of CMI: BSA (1:1, 1:2, 1:5, 1:8, 1:10). Salt concentration of CMI and BSA mixture at the mass ratio of 1:5 was prepared and then adjusted to 0, 10, 50, 100, and 200 mM by NaCl, respectively, to determine the influence of salt concentration on complexes. The pH values of all mixtures were adjusted by 0.5 M NaOH and HCl solution.

### 3.5. Turbidimetric Measurements

Turbidity was measured by a Brinkman PC 910 colorimeter that was equipped with a 1 cm path length optical probe at the wavelength of 420 nm. The colorimeter was calibrated while using DI water and the obtained turbidity (T) was recorded as 100%. Small amounts of HCl solutions were used to adjust the pH of CMI/BSA mixture. The initial pH was first adjusted to 7.0 and the terminal point is 1.5. The pH was monitored with a Thomas Scientific pH meter (Model 8025, Swedesboro, NJ, USA) calibrated with three standard buffers of pH 4.01, 7.01, and 10.01. The pH of mixture decreased every droplet of HCl solution and the time interval between measurements was fixed at 1 min. The turbidity value was also recorded during the titration process. Controls with only CMI or BSA were also surveyed as the background measurements. All of the measurements were conducted at 25 °C.

### 3.6. Isothermal Titration Calorimetry (ITC) Analysis

Thermodynamic analysis of CMI and BSA interaction at 25 °C was measured while using PEAQ-ITC Micro calorimeter (MicroCal Inc., Northampton, MA, UK). CMI and BSA were dissolved to 0.12 % and 0.1% *w*/*v*, respectively, in different ionic strength buffer, pH 4.0. The buffers were set at 10 mM with NaCl. All of the solutions and buffers with different ionic strength were adjusted to pH 4.00 and pH 2.60 while using HCl (0.25, 0.5, 1 mol/L), respectively. Subsequently, the solutions were stored at 4 ℃ for 12 h. Before the measurements, the CMI and BSA solutions were degassed for 5 min. under vacuum. BSA solution or buffers was filled into the cell (280 μL) of the ITC equipment, while the CMI solution was pumped into the injector stirrer syringe (40 μL). 19 injections method was performed in these experiments. After the first injection of 0.4 μL (not calculated in the final results), portions of CMI solution (0.4 μL) were sequentially injected into the titration cell containing BSA solutions or buffers. The duration of each injection was 4.0 s. Time interval between subsequent injections was 150 sec until 19th potion was completely injected. The stir speed was set at 750 rpm. The heats of dilution were measured while using titration of CMI into buffers and subtracted from the raw experimental data. Heat peaks were integrated while using MicroCal PEAQ-ITC Control Software. Thermodynamic parameters, including binding stoichiometry (N), binding constant (K_a_), enthalpy change (∆H), and entropy change (∆S), were calculated by iterative curve fitting of the binding isotherms using a “one set of sites” model. The Gibbs free energy change (∆G) was calculated using the equation ∆G = ∆H − T∆S. Binding of CMI to BSA is expressed as a function of the molar ratio.

### 3.7. Measurements of the Sizes of Complexes

A BIC 90 Plus particle size analyzer that was equipped with a Brookhaven BI-9000AT digital correlator (Brookhaven Instrument Corp., New York, NY, USA) was used to measure the sizes of soluble complex. Measurements were operated at a fixed scattering angle of 90° with a solid-state laser operating at 660 nm and 30 mW power. The mean diameter of soluble complexes was calculated by fitting the intensity-intensity autocorrelation functions with William-Watts single stretched exponential function followed by Stokes-Einstein equation.

### 3.8. ζ-Potential Measurements

The surface charges of CMI/BSA complexes under different pH values and ionic strengths were determined by the Zetasizer Nano ZS-90 instrument (Malvern Instruments Ltd., Southboro, MA, USA). All of the measurements were performed at 25 ± 1 °C.

### 3.9. Morphology and Visual Observation of CMI/BSA Complexes

#### 3.9.1. Visual Observation of CMI/BSA Complexes

Insoluble and soluble CMI/BSA complexes were prepared at pH = 4.00 and 2.60, respectively. Optical pictures of insoluble and soluble CMI/BSA complexes after storage at room temperature for one week were captured while using digital camera.

#### 3.9.2. Morphology of Insoluble Complexes

Images of insoluble complexes were revealed by optical microscope and scanning electron microscope. The solutions of CMI/BSA with different salts were adjusted to pH 4.0 under magnetic stirring. 1 mL of well-distributed suspension was added to glass slide, captured at a standstill for 30 min., and then observed by the microscope (Nikon Eclipse, TE2000-U, Nikon Corporation, Tokyo, Japan). 

#### 3.9.3. Morphologies of Soluble Complexes

Images of soluble complexes (pH 2.6, 10 mM salt concentration) were obtained by atomic force microscopy (AFM). AFM morphology was obtained by scanning samples on Nanoscope IIIa Multi-Mode atomic force microscope (Veeco Instruments Inc., Santa Barbara, CA, USA) that was equipped with a silicon-etched RTESP7 cantilever (Veeco Nanoprobe, Camarillo, CA, USA). 20 μL of soluble complex solution was dripped onto a mica surface. After adsorption for 20 min., the mica was dried under a stream of nitrogen gas. Tapping mode AFM was used for collecting the images. 

### 3.10. Statistical Analysis

All of the measurements were conducted in triplicates. One-way Analysis of Variance (ANOVA) was performed using SPSS 20 software (SPSS Inc., Chicago, IL, USA).

## 4. Conclusions

In summary, non-ionic inulin was successfully modified to anionic CMI. The effects of pH and ionic strength on the formation of CMI/BSA complexes were investigated. Results showed that CMI could interact with BSA to form high turbidity complexes, which can be explained by the models that were proposed by Overbeek, Veis, Dubin, and Tainaka. A similar tendency in turbidity spectra was observed in CMI/BSA and pectin/BSA complexes, suggesting that the type of substituent groups (-COO^−^) and charge density played an important role in polysaccharide/protein complexes system. Insoluble complexes and nanoparticles were observed in the CMI/BSA system at the third region (from pH_φ1_ to pH_φ2_). The carboxymethylation method provided a short-chain functional neutral polysaccharide the opportunity to participate in polysaccharide/protein complex formation. The derivatives with a fixed degree of substitution could form different shapes of complexes with protein via electrostatic and hydrogen bonding interactions. Additionally, these interactions resulted in a change in the secondary structure of BSA. Different morphologies of CMI/BSA complexes were the results from the competition between electrostatic attraction and entropy effects. Until now, most studies have focused on soluble nanocomplexes generated at pH_c_ and insoluble complexes formed at pH_φ1_. However, little attention has been paid to soluble complexes occurring at pH_φ2_. CMI/BSA nanocomplexes that formed at pH_φ2_ provided a novel delivery system to encapsulate nutraceuticals and flavors for health-promotion purpose in common supplementary food, such as beverage or yogurt. Hence, further researches on stability, rheology, and the applications of these nanoparticles are in progress.

## Figures and Tables

**Table 1:** Sizes and ζ-Potentials of carboxymethyl inulin (CMI)/bovine serum albumin (BSA) nanocomplexes under different concentrations of NaCl at pH = 2.6.

C_NaCl_ (mM)	Size (nm)	PDI	ζ-Potential (mv)
0	227.0 ± 0.8	0.178	6.9 ± 0.2
10	146.8 ± 0.1	0.241	7.5 ± 0.3
50	373.7 ± 7.6	0.299	5.0 ± 0.2
100	300.4 ± 8.4	0.302	-
200	275.5 ± 13.4	0.368	-

Values denote mean ± SD, n = 3.

## Abbreviations

BSA: bovine serum albumin; CMI, carboxymethyl inulin; NMR, nuclear magnetic resonance; FT-IR, fourier transform infrared; GPC-MALLS-RID, Gel permeation chromatography multi-angle laser light scattering; DP, degree of polymerization; Da, dalton; DS, degree of substitution; Mw, molecular weight; pI, isoelectric point; C, concentration; mM, millimole; r, ratio; HCl, hydrochloric acid; NaCl, Sodium chloride; DLS, dynamic light scattering; AFM, atom force microscopy; PDI, dispersibility index; NaOH, sodium hydroxide; D_2_O, deuteroxide; DI water, distilled water; T, turbidity.
